# Neonatal Candidemia: Clinical Characteristics and Predictors of 28-Day Mortality

**DOI:** 10.3390/jof12080599

**Published:** 2026-08-12

**Authors:** Hatice Turgut, Ramazan Özdemir

**Affiliations:** Division of Neonatology, Department of Pediatrics, Faculty of Medicine, Inonu University, Malatya 44280, Turkey

**Keywords:** candidemia, intensive care units, neonatal, milk, human, mortality

## Abstract

Neonatal candidemia remains a major cause of morbidity and mortality in neonatal intensive care units, particularly among preterm and very-low-birth-weight infants. This retrospective cohort study evaluated the clinical characteristics, microbiological profiles, and factors associated with 28-day all-cause mortality in neonates with culture-proven candidemia admitted to a tertiary neonatal intensive care unit between January 2011 and December 2025. Demographic, clinical, microbiological, treatment, feeding, and outcome data were collected from medical records, and factors associated with mortality were analyzed using univariate and multivariable Firth-penalized logistic regression. A total of 48 neonates were included, with a median gestational age of 28 weeks (IQR 25–36) and a median birth weight of 967.5 g (IQR 765–2400). The incidence of candidemia was 0.4%. *Candida albicans* was the most frequently isolated species (66.7%). The 28-day all-cause mortality rate was 47.9% (23/48). Non-survivors had lower gestational age and birth weight and developed candidemia earlier than survivors. In the univariable analysis, breast milk exposure before the onset of candidemia was significantly more common among survivors than among nonsurvivors and should be considered a hypothesis-generating finding. In the multivariable analysis, inotropic support (aOR 14.69, 95% CI 2.66–124.74; *p* = 0.001) and thrombocytopenia (aOR 5.91, 95% CI 1.36–34.44; *p* = 0.017) were independently associated with 28-day all-cause mortality. In this cohort, inotropic support and thrombocytopenia were independently associated with 28-day all-cause mortality, whereas the association between breast milk exposure and survival was limited to the univariable analysis.

## 1. Introduction

Neonatal candidemia remains among the important causes of morbidity and mortality in neonatal intensive care units (NICUs). Bloodstream infections caused by *Candida* species occur less frequently than bacterial infections; however, they are associated with higher mortality and long-term neurodevelopmental impairment [1,2]. In the literature, mortality rates related to neonatal invasive candidiasis have been reported to range between 21% and 32%, and this rate may reach as high as 40–50%, particularly for very-low-birth-weight (VLBW) and extremely low-birth-weight (ELBW) infants [3,4].

Preterm neonates are at high risk for invasive candidiasis because of immune system immaturity and impaired skin and mucosal barrier function. Infants with a gestational age of <32 weeks and a birth weight of <1000 g constitute the highest-risk group [5,6]. In this vulnerable population, the use of central venous catheters, invasive mechanical ventilation, total parenteral nutrition (TPN), necrotizing enterocolitis, abdominal surgery, and exposure to broad-spectrum antibiotics have been identified as the major risk factors. Broad-spectrum antibiotics are reported to disrupt the intestinal microbiota, thereby promoting *Candida* overgrowth and translocation, while invasive devices facilitate the entry of *Candida* species into the bloodstream [1,2,5].

The epidemiological characteristics of neonatal candidemia, *Candida* species distribution, antifungal treatment approaches, and predictors of mortality may vary across centers; therefore, center-specific data are important for the development of clinical management strategies. Although numerous studies have investigated the epidemiology, risk factors, and outcomes of neonatal candidemia, the potential contribution of nutritional factors has received relatively little attention. Human milk contains multiple bioactive components with immunomodulatory and antimicrobial properties and is associated with a reduced risk of several infectious morbidities in preterm infants [7]. However, data concerning nutritional factors, particularly the potential association between breast milk exposure and outcomes in patients with neonatal candidemia, remain limited.

Human milk contains numerous bioactive components, including immunoglobulins, lactoferrin, human milk oligosaccharides, and antimicrobial peptides, which contribute to immune maturation and protection against neonatal infections. Previous studies have demonstrated that human milk is associated with a reduced risk of several infectious morbidities in preterm infants. However, evidence regarding its potential association with outcomes in neonatal candidemia remains limited. Therefore, feeding characteristics, including breast milk exposure before candidemia onset, were included among the variables evaluated in this study.

In this study, we aimed to evaluate the demographic, clinical, microbiological, and treatment characteristics of patients with culture-confirmed candidemia followed in a tertiary NICU; to identify risk factors associated with 28-day mortality; and to investigate the relationship between breastfeeding and clinical outcomes.

## 2. Materials and Methods

### 2.1. Study Design and Setting

This retrospective single-center cohort study was conducted in the NICU of a tertiary university hospital between January 2011 and December 2025. Our unit is a regional referral center providing advanced diagnostic, therapeutic, and intensive care services for both neonates born at our center and those referred from external institutions.

### 2.2. Study Population

Neonates admitted to the NICU between January 2011 and December 2025 with at least one positive blood culture for Candida spp. were eligible for the study. Only the first episode of culture-confirmed candidemia was included, and duplicate positive blood cultures obtained during the same candidemia episode were excluded. The onset of candidemia was defined as the date of the first positive blood culture.Complete clinical records, microbiological data, and 28-day follow-up information were available for all included patients.

The primary outcome of the study was 28-day all-cause mortality. Cases were compared according to 28-day survival status after candidemia onset and categorized as survivors and nonsurvivors.

### 2.3. Data Collection and Variables

Clinical, microbiological, treatment, and outcome data were retrospectively extracted from the hospital electronic medical records and microbiology laboratory database and reviewed by the investigators. Demographic, perinatal, clinical, microbiological, treatment-related, and laboratory data were retrospectively obtained from the hospital electronic medical records system. The demographic and perinatal variables included gestational age, birth weight, sex, birth weight <1000 g and <1500 g, prematurity (<37 weeks), mode of delivery, antenatal steroid exposure, premature rupture of membranes, and maternal history of infection.

Clinical, microbiological, treatment-related, and laboratory variables were also collected retrospectively from the medical records (Table 1). The duration of mechanical ventilation, central venous catheterization, total parenteral nutrition; time from the first positive blood culture to the initiation of antifungal therapy (evaluated only in treated patients); and time from candidemia onset to death (evaluated only among non-survivors) were recorded in days.

The results of the microbiological evaluation included the *Candida* species isolated from blood cultures and the initial intravenous antifungal therapies administered. Laboratory parameters analyzed at the onset of candidemia included white blood cell count, hemoglobin level, platelet count, C-reactive protein (CRP), and interleukin-6 (IL-6) level.

### 2.4. Definitions

Candidemia was defined as the isolation of *Candida* spp. in at least one blood culture in the presence of clinical signs of infection. The onset of candidemia was defined as the day on which the first positive blood culture was obtained. Repeated *Candida* isolations belonging to the same infectious episode were not considered separate episodes.

Concomitant bacterial sepsis was defined as the presence of a culture-confirmed bacterial infection during the candidemia episode. Prior antibiotic exposure was defined as antibiotic treatment for at least 7 days before the onset of candidemia. Antifungal prophylaxis consisted of oral nystatin (100,000 U), administered to extremely low-birth-weight infants (<1000 g) and other neonates considered at high risk for invasive candidiasis, in accordance with the unit’s standard clinical protocol, which remained unchanged throughout the 15-year study period.

Full enteral feeding was defined as achieving an enteral intake of ≥120 mL/kg/day. Breast milk exposure was defined as any maternal breast milk received before the onset of candidemia. Donor breast milk was not used during the study period. Detailed information regarding the amount, duration, and exclusivity of breastfeeding was not consistently available from the medical records. Major surgical conditions were defined as the presence of congenital or acquired surgical pathologies requiring surgical intervention during the neonatal period.

The presence of a central venous catheter (CVC), mechanical ventilation, and total parenteral nutrition were recorded as exposure variables if they were present for at least 72 h before the onset of candidemia. Full enteral feeding was defined as the achievement of full enteral nutrition at least 72 h before the onset of candidemia. Inotropic support, thrombocytopenia, and concomitant bacterial bloodstream infection were defined on the basis of findings present at the time of the first positive blood culture for *Candida* species.

The primary outcome of the study was defined as all-cause mortality occurring within 28 days after the onset of candidemia.

### 2.5. Microbiological Methods

Blood culture samples were obtained aseptically from neonates with suspected sepsis as part of routine clinical care and processed in the hospital microbiology laboratory using the BACT/ALERT^®^ 3D automated blood culture system (bioMérieux, Marcy-l’Étoile, France). Blood culture collection and processing followed the standard procedures routinely applied in our neonatal intensive care unit throughout the study period.

Yeast isolates recovered from positive blood cultures were identified to the species level using matrix-assisted laser desorption ionization time-of-flight mass spectrometry (MALDI-TOF MS; bioMérieux, Marcy-l’Étoile, France). Repeated *Candida* isolates recovered from the same patient during a single infectious episode were considered part of the same episode, and only the first episode of culture-confirmed candidemia was included in the analysis.

Antifungal susceptibility testing was performed according to the routine laboratory methods in use during the study period. Etest methodology was used during the early study period; VITEK^®^ 2 (bioMérieux, Marcy-l’Étoile, France) was used between 2022 and 2024; and the Diagnostics broth microdilution system has been used since 2025. The antifungal agents tested included fluconazole, voriconazole, amphotericin B, caspofungin, and micafungin. Minimum inhibitory concentrations (MICs) were interpreted according to the European Committee on Antimicrobial Susceptibility Testing (EUCAST) clinical breakpoints applicable at the time of testing. Susceptibility testing results were available for all included isolates. However, because laboratory practices changed over the 15-year study period, not all antifungal agents were tested for every isolate.

### 2.6. Statistical Analysis

Statistical analyses were performed using IBM SPSS Statistics version 26.0 (IBM Corp., Armonk, NY, USA) and R software 4.5.2 (R Foundation for Statistical Computing, Vienna, Austria). The distribution of continuous variables was assessed using the Kolmogorov–Smirnov and Shapiro–Wilk tests. Continuous variables are presented as medians and interquartile ranges (IQRs), whereas categorical variables are presented as frequencies and percentages. Comparisons between survivors and non-survivors were performed using the Mann–Whitney U test for continuous variables and the chi-square test or Fisher’s exact test, as appropriate, for categorical variables.

Crude associations with 28-day mortality were evaluated using univariable logistic regression analyses. Owing to the limited sample size (48 patients) and the relatively small number of mortality events (23 deaths), Firth-penalized logistic regression was used for multivariable analysis to reduce small-sample bias and avoid separation. No automated variable-selection procedure was applied. Instead, candidate variables were selected according to the results of the univariable logistic regression analyses, together with their clinical relevance and biological plausibility. To minimize overfitting, the number of variables included in the multivariable model were intentionally restricted.

Variables identified as significant in the univariable logistic regression analyses (breast milk feeding, maternal infection, respiratory distress syndrome, mechanical ventilation, thrombocytopenia, inotropic support, and postnatal age at candidemia onset) were considered candidate predictors. Gestational age and birth weight were also considered because of their established biological relevance. The dependent variable was 28-day mortality, and the final multivariable model included inotropic support, platelet count <100,000/µL, and postnatal age at candidemia onset as independent variables. Variable selection was guided by clinical relevance and biological plausibility, with variables representing baseline vulnerability, early nutritional exposure, and disease severity considered during model development. To minimize model overfitting and redundancy among clinically related variables, mechanical ventilation was not retained in the final multivariable model. Potential collinearity among clinically related variables was considered during model development to avoid redundancy in the final model.

No missing data were observed for the variables included in the multivariable analysis. Firth logistic regression was performed using the logistf function from the logistf package in R, and 95% confidence intervals were estimated using profile penalized likelihood. Odds ratios (ORs) with corresponding 95% confidence intervals (CIs) are reported. All statistical tests were two-sided, and a *p* value < 0.05 was considered statistically significant. Given the limited sample size and number of outcome events, the multivariable analysis should be interpreted as exploratory.

### 2.7. Sample Size

Because of the retrospective cohort design of the study, no a priori sample size calculation was performed. All consecutive neonates who met the eligibility criteria and were diagnosed with candidemia during the study period were included in the analysis.

### 2.8. Ethical Considerations

 The study was conducted in accordance with the Declaration of Helsinki and was approved by the Institutional Clinical Research Ethics Committee(Approval No.: 10021/2026; Date: 21 April 2026). The requirement for informed consent was waived because of the retrospective design of the study. All the data were anonymized prior to analysis, and patient confidentiality was strictly maintained.

## 3. Results

**Figure 1:**
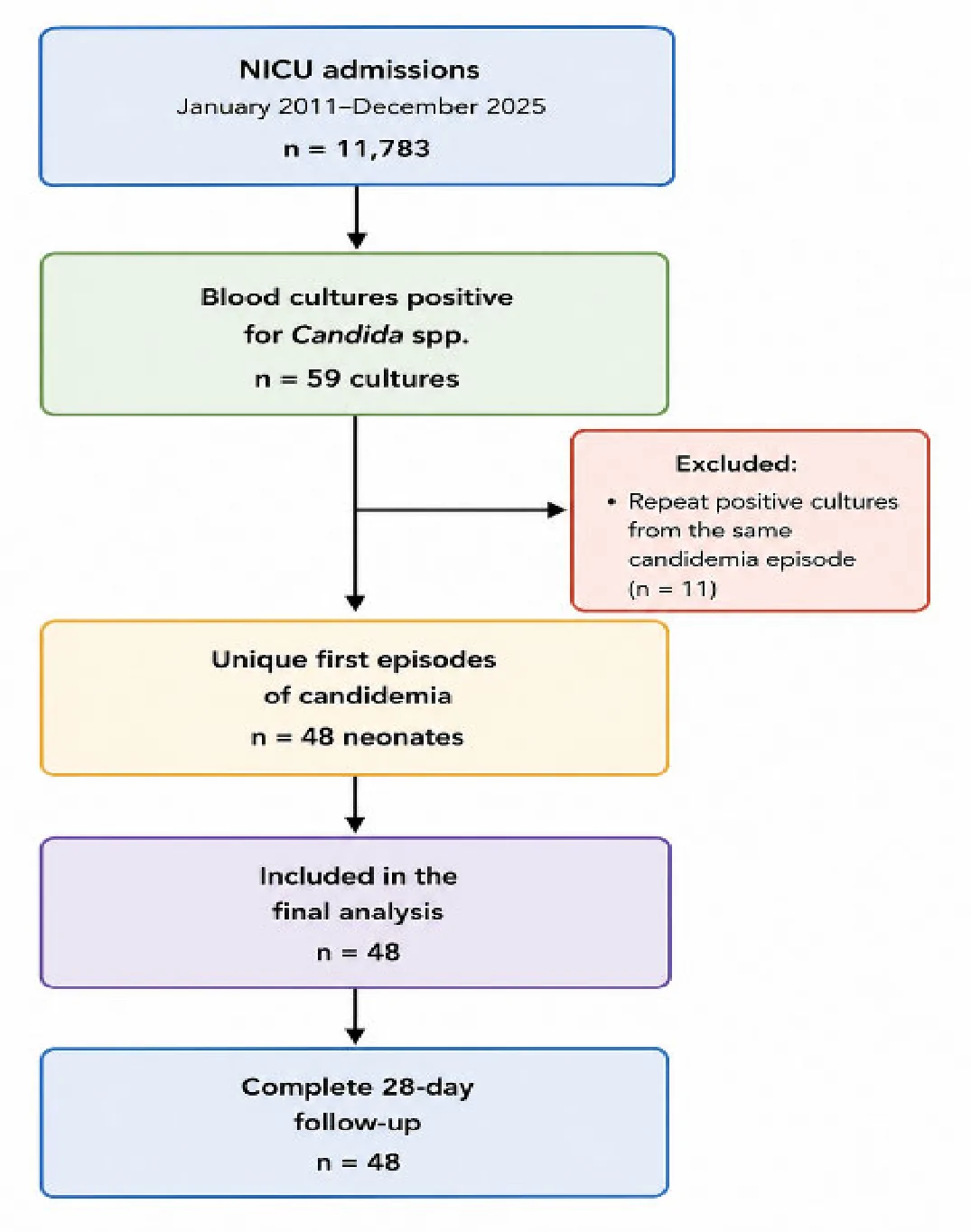
Flow diagram of patient selection.

During the study period, a total of 11,783 neonates were admitted to and monitored in a tertiary NICU, with an overall mortality rate of approximately 6%. A total of 59 blood cultures yielded *Candida* spp.; however, 11 positive cultures represented duplicate cultures from the same candidemia episode and were therefore excluded. Consequently, 48 neonates with a first episode of candidemia were included in the final analysis (Figure 1). This corresponded to an incidence of 4 cases per 1000 admissions (0.4%). The 28-day mortality rate among infants with candidemia was 47.9%.

A total of 48 neonates with candidemia were included in the study. The median gestational age was 28 weeks (IQR: 25–36), and the median birth weight was 967.5 g (IQR: 765–2400). In the cohort, 75% were preterm, and 60.4% had a birth weight of <1500 g. Candidemia was diagnosed at a median postnatal age of 21 days (IQR: 10–32.5) (Table 1).

A substantial proportion of patients required intensive-care-related interventions; mechanical ventilation was used in 58.3% of cases, central venous catheters were present in 70.8%, and TPN was administered in 79.2%. Concomitant culture-confirmed bacterial sepsis was identified in 22.9% of the patients, while previous antibiotic exposure was notably high (95.8%) (Table 1).

Microbiological evaluation revealed that the most frequently isolated pathogen was *Candida albicans* (66.7%), followed by *Candida parapsilosis* (18.7%) and *Candida glabrata* (14.6%). With respect to antifungal treatment, fluconazole was the most commonly used agent (47.9%), followed by amphotericin B (31.3%). In 18.8% of the patients, antifungal therapy could not be initiated because blood culture results confirming candidemia were available only after death (Table 1). Among the remaining 39 neonates who received antifungal therapy, the median time from the first positive blood culture to the initiation of antifungal therapy was 2 days (IQR, 2–3).

Among the 48 neonates with candidemia, 23 (47.9%) died during the 28-day follow-up period. Compared with survivors, nonsurvivors had lower gestational ages (*p* = 0.046) and birth weights (*p* = 0.032). A maternal history of infection was more common among nonsurvivors (*p* = 0.020), and candidemia occurred earlier in the postnatal period (*p* = 0.010). Respiratory distress syndrome (*p* = 0.021), the need for inotropic support (*p* < 0.001), mechanical ventilation (*p* = 0.001), and a longer duration of mechanical ventilation (*p* < 0.001) were also more common among nonsurvivors. Platelet counts were lower (*p* = 0.006), whereas breast milk feeding was more common in survivors (*p* = 0.021). No significant differences were observed for the remaining clinical or laboratory variables (Table 2).

The distribution of *Candida* species did not differ significantly between survivors and nonsurvivors (Fisher’s exact test, *p* = 0.629). Although *Candida albicans* was more frequently isolated among nonsurvivors (73.9% vs. 60.0%), no significant association between *Candida* species and 28-day mortality was observed (Table 3).

Fluconazole resistance was observed predominantly among *Candida glabrata*, whereas amphotericin B resistance was detected only in two *Candida albicans* isolates. Antifungal susceptibility according to *Candida* species is summarized in Supplementary Table S1.

Firth-penalized logistic regression analysis was performed to evaluate factors associated with 28-day mortality (Table 4). In the univariable analysis, breast milk consumption was associated with lower odds of mortality (OR: 0.26; 95% CI: 0.07–0.83; *p* = 0.023). However, because of the limited sample size and the predefined multivariable modeling strategy, breastfeeding was not retained in the final adjusted model. In contrast, a maternal history of infection, respiratory distress syndrome, the need for mechanical ventilation, the need for inotropic support, thrombocytopenia, and earlier postnatal onset of candidemia were associated with higher odds of mortality in the univariable analysis. In the multivariable analysis, only inotropic support (adjusted OR: 14.69; 95% CI: 2.66–124.74; *p* = 0.001) and thrombocytopenia (adjusted OR: 5.91; 95% CI: 1.36–34.44; *p* = 0.017) remained independently associated with 28-day mortality (Table 4).

## 4. Discussion

### 4.1. Mortality Rate and Burden of Prematurity

Neonatal candidiasis remains an important cause of morbidity and mortality, particularly among ELBW infants. In the literature, the incidence of candidemia has been reported to range between 0.26% and 1.6% in the overall NICU population, while this rate increases to 7–9% among ELBW infants [1,8,9,10]. In our cohort, which included a high proportion of ELBW (52.1%) and VLBW (60.4%) infants, the overall incidence of candidemia was 0.4%, while the mortality rate of 47.9% may be explained by the high-risk clinical characteristics of this vulnerable population. In the literature, mortality rates associated with fungal sepsis have generally been reported to range from 20% to 38.5%, although they may reach 50% in ELBW cohorts [3,4,8,11]. In a study by Özmen et al., which evaluated 26 neonatal candidemia cases, the incidence of candidemia was reported to be 0.28%, the mortality rate was 38.5%, and the proportion of ELBW infants was 30.8% [6]. Similarly, Tunç et al. reported a candidemia incidence of 1.6% and a mortality rate of 35%, demonstrating a marked increase in mortality among extremely preterm infants [8]. The relatively higher mortality rate observed in our cohort may be related to the greater burden of advanced prematurity and low birth weight in our patient population.

### 4.2. Candida Species Distribution and Clinical Significance

Although *Candida albicans* remains the most common pathogen in neonatal candidemia, a marked increase in nonalbicans *Candida* species has been reported in recent years [2,6]. This increase is clinically important because antifungal susceptibility patterns may differ among species, and azole resistance in particular may be more frequently observed. Therefore, species-level identification of the causative organism is critical for selecting appropriate empirical antifungal therapy and guiding antifungal resistance management. In the literature, *Candida albicans* has been reported in 35–75% of neonatal candidemia cases [12,13], whereas *Candida parapsilosis* is the most frequently isolated nonalbicans species, accounting for 14–46.2% of cases [6,8]. Previous studies have reported fluconazole resistance rates of 15.4–59% for *Candida parapsilosis*, whereas amphotericin B resistance has been described less frequently, at approximately 7% [6,12]. In our study, *Candida albicans* was also the most frequently isolated pathogen, while nonalbicans *Candida* species accounted for 33.3% of all cases; among these cases, *Candida parapsilosis* was the most common species identified (n = 9, 18.7%). Overall, fluconazole resistance was identified in 5 of 48 isolates (10.4%), predominantly among *Candida glabrata*, whereas amphotericin B resistance was identified in only two *Candida albicans* isolates. These findings highlight the importance of local resistance patterns in guiding empirical antifungal treatment decisions for neonatal candidemia. The absence of a significant association between Candida species distribution and 28-day mortality suggests that mortality may be more closely related to underlying clinical vulnerability than to the species distribution itself.

### 4.3. Timing of Candidemia and Early Mortality

In our study, candidemia was diagnosed in neonates at a median postnatal age of 21 days (10.0–32.5). These findings are consistent with those of previous reports indicating that the majority of candidiasis cases in NICUs present as late-onset sepsis [2,8]. In the literature, the age at onset of candidemia has generally been reported to range between 11 and 20 days, whereas a multicenter NeoOBS study reported a median onset of 16 days [3,5,12].

One notable finding of our study was that candidemia developed significantly earlier in nonsurvivors than in survivors (*p* = 0.010). This may suggest that fungal infections occurring early in life coincide with the period when preterm neonates are immunologically and physiologically most vulnerable, which may contribute to a more severe clinical course and increased mortality. Previous studies have similarly reported that infections occurring in the early postnatal period are associated with increased mortality and tend to follow a more severe course, particularly in VLBW infants [5,6].

One possible explanation for the poor prognosis associated with candidemia developing in the early postnatal period may be the fulminant clinical course of invasive candidiasis. In our study, the median time from candidemia onset to death was 2 days (1.5–4.5), and in 9 patients, the diagnosis could only be confirmed after death on the basis of blood culture results. The occurrence of blood culture confirmation only after death in these patients further underscores the fulminant nature of invasive candidiasis, indicating that the disease may progress before microbiological confirmation can be obtained. These findings suggest that *Candida* infections may begin with nonspecific clinical manifestations and rapidly progress to severe sepsis, septic shock, and death within a short period of time [5]. Similarly, Jantarabenjakul et al. reported that, in invasive candidiasis cases diagnosed postmortem, the median interval between clinical sampling and death was 1 day (0–3) [14]. In addition, Cahan and Deville demonstrated that each 24-h delay in the initiation of antifungal therapy increased the risk of mortality by 10.9% (OR: 1.109; 95% CI: 1.003–1.226) [15]. Our findings support the concept that invasive candidiasis may follow an aggressive and rapidly progressive clinical course in high-risk neonates and highlight the importance of prompt diagnostic evaluation, early empirical antifungal treatment, and close clinical monitoring when candidemia is clinically suspected.

### 4.4. Invasive Interventions and Intensive Care Burden

In NICUs, invasive life-support interventions used to improve survival are among the major risk factors for the development of candidiasis [2,16,17]. The literature reports that these interventions disrupt epidermal and mucosal barrier integrity, creating a portal of entry for Candida species; in particular, biofilm formation on catheter surfaces makes eradication of the infection more difficult. In addition, the composition of TPN is known to promote fungal proliferation and biofilm formation [16,17]. In our study, CVCs were present in 70.8% of patients, TPN was administered in 79.2%, and mechanical ventilation was required in 58.3%. Similarly, previous studies have reported TPN use rates ranging from 95.5–97.7%, CVC presence rates ranging from 75.3–79%, and exposure to mechanical ventilation rates ranging from 56% to 58% [1,3,5,14,18].

In our study population, the median duration of TPN was 8 days, and the median duration of CVC use was 9 days, reflecting the substantial need for prolonged invasive support among neonates with candidemia. Prolonged exposure to these interventions has been reported to facilitate Candida colonization and invasion, particularly through disruption of immature skin and mucosal barrier integrity. Together, these findings highlight the considerable intensive care burden in neonates with candidemia and underscore the importance of minimizing unnecessary invasive device exposure whenever clinically feasible.

### 4.5. Nutrition and Breast Milk Feeding

Previous studies have identified delayed achievement of full enteral feeding as an important risk factor for candidiasis and mortality in the NICU [17]. In our study, only 20.8% of patients had reached full enteral feeding before the onset of candidemia, indicating that delayed establishment of enteral nutrition was common among neonates with candidemia, which is consistent with previous reports [16,17]. In addition to low gestational age and birth weight, the presence of severe surgical conditions—predominantly gastrointestinal surgeries—in 43.8% of cases may have delayed the transition to enteral feeding in our cohort. The resulting increased need for TPN and central venous catheter use reflects the clinical complexity and intensive care requirements of these patients.

When the relationship between feeding type and mortality was evaluated, the rate of breastfeeding was greater among survivors (*p* = 0.021). Univariable logistic regression analysis revealed that breast milk consumption was associated with lower odds of mortality (crude OR: 0.259; 95% CI: 0.074–0.833; *p* = 0.023). This unadjusted association should be considered hypothesis-generating and requires confirmation in larger prospective studies. Moreover, breast milk exposure may also reflect greater clinical maturity, better feeding tolerance, or lower illness severity rather than a direct protective effect. Previous studies have shown that lactoferrin and other immunobiological components present in breast milk support intestinal barrier integrity, regulate the intestinal microbiota, and limit microbial translocation [7].

### 4.6. Factors Associated with 28-Day Mortality

Multivariate regression analysis revealed clinical factors associated with 28-day mortality in patients with neonatal candidemia. Because the univariable analyses were performed without adjustment for multiple comparisons, independent associations with 28-day mortality should be interpreted primarily on the basis of the multivariable regression results. In this model, the need for inotropic support (adjusted OR: 14.69; *p* = 0.001) and thrombocytopenia (adjusted OR: 5.91; *p* = 0.017) remained significantly associated with mortality. Both findings are likely to reflect the severity of the underlying clinical condition rather than represent direct causal determinants of mortality. Accordingly, these variables should be interpreted as severity-associated mortality markers rather than causal predictors. Previous studies have similarly reported that septic shock is among the major determinants of poor prognosis in invasive fungal infections and may contribute to treatment failure [14,19]. Similarly, thrombocytopenia has been consistently associated with severe infection and has been reported more frequently in fatal cases [5,14]. Although gestational age and birth weight differed between survivors and non-survivors in the univariable analyses, they were not independently associated with mortality after multivariable adjustment. These findings may reflect the limited sample size and the stronger influence of acute clinical severity markers in the multivariable model. Taken together, these findings suggest that neonates who require inotropic support or who present with thrombocytopenia constitute a high-risk group that may benefit from closer monitoring and timely clinical intervention.

This study has several limitations. First, owing to its retrospective single-center design, causal inferences are limited, and the generalizability of the findings should be interpreted with caution. Second, the relatively small sample size and limited number of mortality events may have reduced the statistical power of the analyses, particularly in the multivariable model. In addition, because the study was based on retrospective medical records, certain clinical variables—including the amount of enteral feeding, the burden of *Candida* colonization, and the temporal response to antifungal treatment—could not be comprehensively assessed. Furthermore, breast milk exposure was broadly defined as any maternal breast milk received before the onset of candidemia, and detailed information regarding the duration and exclusivity of breast milk feeding was not consistently available. In addition, a small number of postmortem culture-confirmed candidemia cases were included in the mortality analyses, which may have influenced the observed mortality estimates. Furthermore, given the long study period spanning 2011 to 2025, changes in neonatal intensive care practices and microbiological diagnostic approaches over time may have introduced clinical heterogeneity. Finally, mortality was assessed as 28-day all-cause mortality, and deaths directly attributable to candidemia could not be distinguished from those related to underlying comorbidities.

Nevertheless, our study has several important strengths. One of the main strengths of this study is its focus on culture-confirmed neonatal candidemia cases together with the combined evaluation of detailed clinical and microbiological data obtained from tertiary NICUs. The assessment of candidemia onset, invasive interventions, Candida species distribution, antifungal susceptibility patterns, and predictors of mortality within the same cohort provided a comprehensive clinical evaluation. In addition, the evaluation of nutritional characteristics—particularly the association between breast milk consumption and mortality—offers a novel contribution to the literature on neonatal candidemia. The use of Firth-penalized logistic regression to assess independent risk factors associated with mortality further strengthens the findings despite the limited sample size. Overall, we believe that our study contributes to a better understanding of the clinical course of neonatal candidemia and may help support the early identification of high-risk patients as well as the development of infection management and nutritional strategies.

## 5. Conclusions

Our findings suggest that mortality in neonatal candidemia patients is more closely associated with clinical severity than with microbiological characteristics. The need for inotropic support and thrombocytopenia were significantly associated with 28-day mortality and should be interpreted as markers of disease severity rather than causal predictors. Although breastfeeding was associated with lower mortality in the univariable analysis, these findings should be considered hypothesis-generating. Further prospective studies are warranted to validate these observations and improve risk stratification in neonatal candidemia patients.

## Figures and Tables

**Table 1:** Baseline demographic, clinical, microbiological, treatment, and laboratory characteristics of neonates with candidemia.

Variables	Overall Cohort (n = 48)
Gestational age, weeks	28 (25–36)
Birth weight, g	967.5 (765–2400)
Male sex, n (%)	20 (41.7)
<1000 g, n (%)	25 (52.1)
<1500 g, n (%)	29 (60.4)
<37 weeks, n (%)	36 (75.0)
Cesarean delivery, n (%)	31 (64.6)
Antenatal steroid exposure, n (%)	16 (33.3)
Maternal history of infection, n (%)	25 (52.1)
Premature rupture of membranes, n (%)	11 (22.9)
Postnatal age at candidemia onset, days	21 (10.0–32.5)
Respiratory distress syndrome, n (%)	23 (47.9)
Surgical condition, n (%)	21 (43.8)
Inotropic support, n (%)	17 (35.4)
Mechanical ventilation, n (%)	28 (58.3)
Central venous catheter, n (%)	34 (70.8)
Total parenteral nutrition, n (%)	38 (79.2)
TPN duration, days	8 (4.0–15.0)
Mechanical ventilation duration, days	4 (0.0–11.0)
Central venous catheter duration, days	9 (4.0–12.0)
Full enteral feeding, n (%)	10 (20.8)
Breast milk feeding, n (%)	29 (60.4)
Postnatal steroid use, n (%)	4 (8.3)
Antifungal prophylaxis, n (%)	23 (47.9)
Concurrent culture-proven bacterial sepsis, n (%)	11 (22.9)
Prior antibiotic use, n (%)	46 (95.8)
Cand ida species, n (%)	32 (66.7)
*Candida albicans*	9 (18.7)
*Candida parapsilosis*	7 (14.6)
*Candida glabrata*	
Initial antifungal treatment, n (%)	23 (47.9)
Fluconazole	15 (31.3)
Amphotericin B	1 (2.1)
Voriconazole	9 (18.8)
No antifungal treatment before death	
Time from first positive blood culture to antifungal initiation, days †	2 (2–3)
Days from candidemia onset to death *	2 (1.5–4.5)
28-day mortality, n (%)	23 (47.9)

Data are presented as medians (interquartile ranges) or n (%). The exposure variables were defined as described in the Methods section. † Calculated only among patients who received antifungal therapy (n = 39). Patients who were diagnosed with candidemia only after death (n = 9) were excluded from this analysis. * Days from candidemia onset to death were calculated for nonsurvivors only (n = 23).

**Table 2:** Comparisons of Demographic, Perinatal, Clinical, and Laboratory Characteristics between Survivors and Nonsurvivors.

Variables	Survivors (n = 25)	Non-Survivors (n = 23)	*p* Value
Gestational age, weeks *	30.0 (27.0–36.0)	26.0 (24.5–34.5)	0.046
Birth weight, g *	1430 (905–2500)	845 (645–1975)	0.032
Male sex, n (%)	12 (48.0)	8 (34.8)	0.353
<1500 g, n (%)	13 (52.0)	16 (69.6)	0.214
<37 weeks, n (%)	19 (76.0)	17 (73.9)	0.868
Cesarean delivery, n (%)	16 (64.0)	15 (65.2)	0.930
Antenatal steroid exposure, n (%)	7 (28.0)	9 (39.1)	0.414
Maternal history of infection, n (%)	9 (36.0)	16 (69.6)	0.020
Premature rupture of membranes (PROM), n (%)	6 (24.0)	5 (21.7)	0.852
Postnatal age at candidemia onset, days *	31 (14–48)	14 (7–24)	0.010
Respiratory distress syndrome, n (%)	8 (32.0)	15 (65.2)	0.021
Surgical condition, n (%)	13 (52.0)	8 (34.8)	0.230
Inotropic support, n (%)	2 (8.0)	15 (65.2)	<0.001
Mechanical ventilation, n (%)	9 (36.0)	19 (82.6)	0.001
Mechanical ventilation duration, days *	0.0 (0.0–3.0)	10.0 (4.0–18.0)	<0.001
Central venous catheter, n (%)	16 (64.0)	18 (78.3)	0.278
Central venous catheter duration, days *	7.5 (0.0–12.0)	9.0 (6.0–12.0)	0.192
Total parenteral nutrition, n (%)	17 (68.0)	21 (91.3)	0.075
TPN duration, days *	8.5 (3.0–15.0)	8.0 (5.5–17.0)	0.724
Full enteral feeding, n (%)	7 (28.0)	3 (13.0)	0.292
Breast milk feeding, n (%)	19 (76.0)	10 (43.5)	0.021
Postnatal steroid use, n (%)	3 (12.0)	1 (4.3)	0.610
Antifungal prophylaxis, n (%)	9 (36.0)	14 (60.9)	0.085
Concurrent culture-proven bacterial sepsis, n (%)	7 (28.0)	4 (17.4)	0.382
Prior antibiotic use, n (%)	23 (92.0)	23 (100.0)	0.490
WBC (×10^3^/µL) *	13.3 (8.2–26.2)	22.4 (11.5–40.0)	0.121
Hemoglobin (g/dL) *	11.4 (9.3–13.4)	11.3 (9.1–13.0)	0.947
Platelet (×10^3^/µL) *	145.0 (93.5–229.5)	50.0 (29.0–94.0)	0.006
CRP (mg/L) *	6.2 (0.6–10.6)	2.1 (0.7–6.6)	0.293
IL-6 (pg/mL) *	180 (111–447)	276 (179–865)	0.356

* Data are presented as medians (interquartile ranges [IQRs]) or n (%). The exposure variables were defined as described in the Methods section. Abbreviations: PROM, premature rupture of membranes; TPN, total parenteral nutrition; CRP, C-reactive protein; IL-6, interleukin-6; WBC, white blood cell count. IL-6 measurements were available for 17 survivors and 16 nonsurvivors.

**Table 3:** Candida species according to 28-day survival.

Candida Species	Survivors (n = 25), n (%)	Nonsurvivors (n = 23), n (%)	Total (n = 48), n (%)	*p* Value
*Candida albicans*	15 (60.0)	17 (73.9)	32 (66.7)	
*Candida parapsilosis*	6 (24.0)	3 (13.0)	9 (18.8)	
*Candida glabrata*	4 (16.0)	3 (13.0)	7 (14.6)	
**Total**	**25 (100)**	**23 (100)**	**48 (100)**	**0.629** *

Data are presented as n (%). Percentages were calculated within each survival group. * *p* value was calculated using Fisher’s exact test.

**Table 4:** Univariable and multivariable Firth-penalized logistic regression analysis for mortality.

Variable	Crude OR (95% CI)	*p* Value	Adjusted OR (95% CI)	*p* Value
Breast milk feeding	0.26 (0.07–0.83)	0.023		-
Birth weight (per 100 g increase)	0.96 (0.90–1.02)	0.265		-
Gestational age (per week increase)	0.92 (0.83–1.02)	0.119		-
Inotropic support	17.14 (4.17–101.48)	<0.001	14.69 (2.66–124.74)	0.001
Maternal history of infection	3.82 (1.22–12.99)	0.021		-
Mechanical ventilation	7.52 (2.19–30.46)	0.001		-
Platelet count <100,000/µL	6.17 (1.83–23.62)	0.003	5.91 (1.36–34.44)	0.017
Postnatal age at candidemia onset	0.95 (0.91–0.99)	0.008	0.99 (0.94–1.05)	0.764
Respiratory distress syndrome	3.75 (1.20–12.67)	0.023		-

Abbreviations: OR, odds ratio; CI, confidence interval. Univariable and multivariable associations with 28-day mortality were evaluated using Firth-penalized logistic regression. Variables not retained in the final multivariable model are indicated by a dash (-) because the final model was intentionally restricted to minimize overfitting.

## Data Availability

The datasets generated during and/or analyzed during the current study are not publicly available because of patient confidentiality and institutional restrictions but are available from the corresponding author on reasonable request.

## Supplementary Materials

The following supporting information can be downloaded at https://www.mdpi.com/article/10.3390/jof12080599/s1.

## Abbreviations

The following abbreviations are used in this manuscript:

CRP	C-reactive protein
CVC	Central venous catheter
ELBW	Extremely low birth weight
IL-6	Interleukin-6
NICU	Neonatal intensive care unit
PROM	Premature rupture of membranes
TPN	Total parenteral nutrition
VLBW	Very low birth weight
