# Supplementary material for: Neonatal Candidemia: Clinical Characteristics and Predictors of 28-Day Mortality
Source: J Fungi (Basel). 2026 Aug 12;12(8):599. doi: 10.3390/jof12080599 (PMC13514253; doi:10.3390/jof12080599)
Supplement: Supplementary file 1 [file jof-12-00599-s001.zip › jof-4391648-supplementary.pdf]

Supplementary Table S1. Antifungal susceptibility profiles according to *Candida* species

Candida species	Fluconazole Resistance, n/N (%)	Amphotericin B Resistance, n/N (%)	Voriconazole Resistance, n/N (%)	Micafungin Resistance, n/N (%)	Caspofungin Resistance, n/N (%)
C. albicans	0/32 (0.0)	2/32 (6.3)	0/14 (0.0)	0/9 (0.0)	0/9 (0.0)
C. parapsilosis	1/9 (11.1)	0/9 (0.0)	0/4 (0.0)	0/1 (0.0)	0/1 (0.0)
C. glabrata	4/7 (57.1)	0/7 (0.0)	0/3 (0.0)	0/4 (0.0)	0/4 (0.0)
**Total**	**5/48 (10.4)**	**2/48 (4.2)**	**0/21 (0.0)**	**0/14 (0.0)**	**0/14 (0.0)**

Data are presented as the number of resistant isolates divided by the number of isolates tested for each antifungal agent. Because laboratory methods evolved during the 15-year study period, not all antifungal agents were tested for every isolate. Minimum inhibitory concentrations (MICs) were interpreted according to the European Committee on Antimicrobial Susceptibility Testing (EUCAST) clinical breakpoints applicable at the time of testing.
